# Prevalence and Risk Factors of Respiratory Syncytial Virus in Children under 5 Years of Age in the WHO European Region: A Systematic Review and Meta-Analysis

**DOI:** 10.3390/jpm11050416

**Published:** 2021-05-15

**Authors:** Nora Suleiman-Martos, Alberto Caballero-Vázquez, Jose Luis Gómez-Urquiza, Luis Albendín-García, Jose Luis Romero-Béjar, Guillermo A. Cañadas-De la Fuente

**Affiliations:** 1 Faculty of Health Sciencies, University of Granada, Cortadura del Valle S/N, 51001 Ceuta, Spain; norasm@ugr.es; 2 Diagnostic Lung Cancer Unit, Broncopleural Techniques and Interventional Pulmonology Department, Hospital Universitario Virgen de las Nieves, 18014 Granada, Spain; alberto.caballero.sspa@juntadeandalucia.es; 3 Faculty of Health Sciencies, University of Granada, Avenida Ilustración, 60, 18016 Granada, Spain; jlgurquiza@ugr.es (J.L.G.-U.); gacf@ugr.es (G.A.C.-D.l.F.); 4 Granada-Metropolitan District, Andalusian Health Service, Avenida del Sur, 11, 18014 Granada, Spain; lualbgar1979@ugr.es; 5 Department of Statistics and Operational Research, University of Granada. Av. Fuentenueva, 18071 Granada, Spain

**Keywords:** respiratory syncytial virus, risk factors, prevalence, children, epidemiology, meta-analysis

## Abstract

A respiratory syncytial virus (RSV) is the major cause of respiratory tract infection in children under 5 years. However, RSV infection in the European Region of the World Health Organization has not been systematically reviewed. The aim was to determine the prevalence and factors associated with RSV in children under 5 years of age in European regions. A systematic review and meta-analysis was performed. CINAHL, Medline, LILACS, ProQuest, SciELO, and Scopus databases were consulted for studies published in the last 5 years, following Preferred Reporting Items for Systematic Reviews and Meta-analysis guidelines. The search equation was “respiratory syncytial virus AND (newborn OR infant OR child) AND (prevalence OR risk factors)”. Studies reporting the prevalence of RSV were eligible for inclusion in the meta-analysis. A total of 20 articles were included. The meta-analytic prevalence estimation of RSV, with a sample of n = 16,115 children, was 46% (95% CI 34–59%). The main risk factors were age, male gender, winter season, and environmental factors such as cold temperatures, higher relative humidity, high concentrations of benzene, exposure to tobacco, and living in urban areas. Robust age-specific estimates of RSV infection in healthy children should be promoted in order to determine the optimal age for immunization. In addition, it is necessary to analyse in greater depth the potentially predictive factors of RSV infection, to be included in prevention strategies.

## 1. Introduction

Respiratory syncytial virus (RSV) is a single-stranded RNA virus that belongs to the Paramyxoviridae family, affecting respiratory epithelial cells and presenting two subtypes, RSV A (the most severe form of presentation) and RSV B [1].

It is one of the main causes of acute lower respiratory tract infection (ALRI), particularly in children under one year of age [2]. Worldwide, it is estimated that there are 33 million cases of ALRI per year associated with RSV in children under 5 years of age [3] of which 3.2 million cases require hospitalization [4]. In addition, RSV is one of the main causes of mortality with about 60 thousand deaths per year throughout the world in children under 5 years of age [5].

RSV is characterized by being seasonal, whose infection rate peaks in the cold season in temperate climates [6]. Primary infection from 6 months to 2 years is usually symptomatic, with around 40% of infections presenting bronchiolitis and pneumonia. The incubation period of the infection ranges from 3 to 5 days, and the clinical presentation of RSV can vary according to age, with the most common symptoms being rhinorrhoea, nasal congestion, cough, fever or respiratory distress [7]. Up to 40% of infants progress to ALRI with coughing and wheezing, which range in severity from mild to moderate illness to life-threatening respiratory failure [8]. In addition, the consequences can be negative, especially in children with underlying diseases (prematurity, lung disease, congenital heart disease, congenital or acquired immunodeficiency, or Down syndrome) [9,10,11].

Currently, the drug of choice as prophylaxis to prevent RSV infection is palivizumab, being recommended for high-risk population [12]. However, the high rate of hospitalization and mortality has produced a growing interest in new vaccines and therapies against RSV, in order to include a larger target population such as infants, pregnant women, or the elderly population [13,14]. In the past decade, 10 vaccines and 11 therapeutic agents in active clinical trials have been developed, noting that maternal vaccination is particularly relevant [13]. New treatments have also been developed such as Ribavirin, although not routinely recommended in light of limited evidence of benefit [15]. Nevertheless, the treatment and prophylaxis options are still limited.

In 2015, the World Health Organization (WHO) indicated a high priority on establishing global RSV disease surveillance systems, as well as robust age-specific estimations in order to determine the optimal age for immunization [16]. However, data on the prevalence of RSV and the burden of disease in healthy children are scarce, as most studies are conducted only in high-risk groups [17,18,19].

Specifically, in Europe, there are few studies on RSV infection. Some studies have investigated the seasonality and geography of RSV in European countries [20]. However, to date, there is no systematic review that evaluates the prevalence and risk factors of RSV in healthy children in the WHO European Region. Therefore, and in order to obtain a better understanding of the severity of RSV infection, the objective of this systematic review and meta-analysis is to analyse the prevalence and risk factors of RSV infection in children under 5 years of age.

## 2. Materials and Methods

A systematic review was performed following PRISMA statement (Preferred Reporting Items for Systematic Reviews and Meta-analyses) [21].

### 2.1. Data Sources and Search Strategy

CINAHL, Medline, LILACS, ProQuest, SciELO and Scopus databases were consulted. The search was done in December 2020, using MeSH descriptors and with the following search equation: “respiratory syncytial virus AND (newborn OR infant OR child) AND (prevalence OR risk factors)”.

### 2.2. Study Selection Process

Two authors independently analysed the title and abstract of the articles found, eliminating duplicate studies. Subsequently, the full-texts were reviewed, evaluating them according to inclusion criteria. In case of disagreement, a third author was consulted.

### 2.3. Inclusion and Exclusion Criteria

The inclusion criteria were the following: (1) countries included in the WHO European Region; (2) published in English, French, Spanish, Portuguese, and Italian; (3) published in the last 5 years; (4) children under 5 years of age; (5) gestational age greater than 37 weeks; (6) diagnosis of acute respiratory tract infection (respiratory infection, pneumonia, or bronchiolitis); (7) RSV diagnosis confirmed by laboratory tests; (8) prevalence measurement showing RSV infection positive rate outcome data or providing enough information to calculate the effect size (number of RSV infected patients and total number of patients); and (9) RSV-associated factors.

Articles that did not meet the following criteria were excluded: (1) without a clear diagnosis of RSV, (2) studies that included a high-risk population (with a diagnosis of chronic lung disease, bronchopulmonary dysplasia, cystic fibrosis, or premature infants), (3) with prior prophylaxis (palivizumab or other prevention strategies for RSV infection), and (4) case definition that was not clearly defined.

We did not include studies published before 2015 because seasonality could change and our objective was to report on current global seasonality. There were no restrictions on the healthcare setting or definition of RSV infection.

### 2.4. Data Extraction

Two authors extracted data from all included studies using a data coding form. A third author checked the data for disagreement. The following variables were obtained for each of the articles: (1) author, year of publication, and country; (2) design and study period; (3) age; (4) diagnostic procedure used; (5) sample; (6) place of detection of the case; (7) prevalence; and (8) risk factors.

To assess the reliability of the data coding by the researchers, the intraclass correlation coefficient was calculated as 0.98 (minimum = 0.97; maximum = 1). Cohen’s Kappa coefficient used for categorical variables was 0.97 (minimum = 0.96; maximum = 1).

### 2.5. Quality Evaluation and Risk Bias

In order to assess the quality of the studies included in the review, the levels of evidence and grades of recommendation of the Oxford Center for Evidence-based Medicine (OCEBM) were followed [22], as well as the STROBE (Strengthening the Reporting of Observational Studies in Epidemiology) guide [23].

### 2.6. Data Analysis

A random effects meta-analysis was performed to calculate the prevalence of RSV and the corresponding confidence interval. The software used was StatsDirect (StatsDirect Ltd., Cambridge, UK), presenting the results grouped in a forest plot.

Analysis of heterogeneity was performed by measuring I^2^. There was significant heterogeneity if the I^2^ values were greater than 50% [24]. Publication bias was assessed using Egger’s linear regression test and also a sensitivity analysis was performed.

## 3. Results

**Figure 1:**
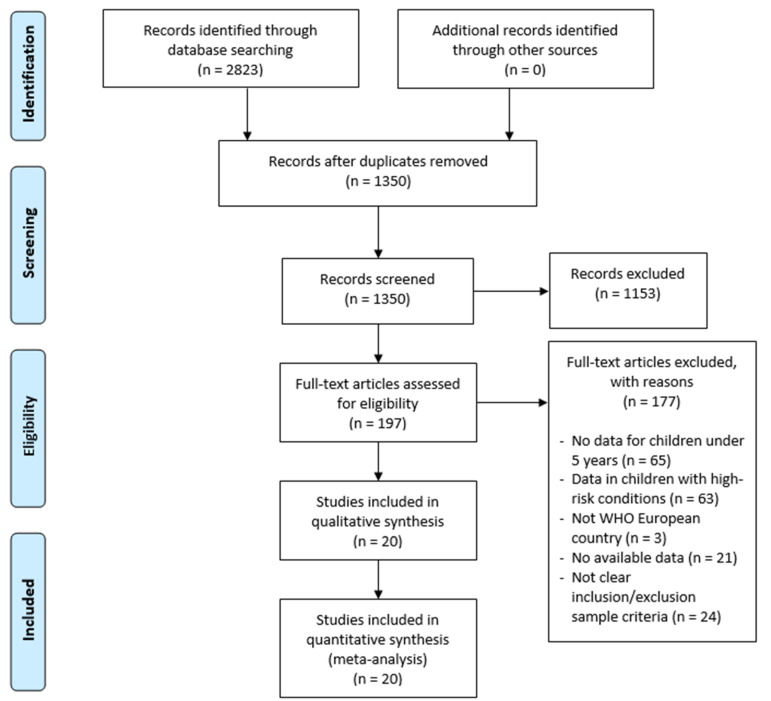
Flow diagram of the selection process.

The initial search strategy identified 2823 references. After reading the title and abstract, 1153 were eliminated. After reading the full text, a total of 177 articles were eliminated. The selection of articles is reflected in Figure 1.

### 3.1. Characteristics of Included Studies

The sample consisted of a total of n = 16,115 children. Most of the studies were cohort studies (75%), followed by cross-sectional studies (20%) and cases and controls (5%). Most of the studies were conducted in Italy (*n* = 6), followed by the Netherlands (*n* = 3) and Bulgaria (*n* = 3). Regarding methodological quality, all studies had an adequate level of quality. The evaluation and characteristics of the studies are represented in Table 1.

### 3.2. Risk Factor (Age)

The hospitalization rate presents an inversely proportional relationship to age, reaching a maximum in the first months of life [29,36]. For some, in the first 6 months, there is a greater RSV infection [32,34], although other authors find that the frequency of RSV-positives increases after 5 months of age compared to those from 0 to 4 months [30]. However, for other authors, the highest number of cases is between the first and third year of life [35,42]. See Table 2.

### 3.3. Risk Factor (Gender and Weight at Birth)

Regarding gender, there is a relationship between male gender and RSV infection, presenting a higher percentage of infection [30,33,34,39], although other authors do not find any relationship [38].

Low birth weight is also considered a risk factor [36] and more specifically if it is below the third percentile [39], although other authors find no relationship [38,40].

### 3.4. Risk Factor (Seasonality)

The seasonal distribution of RSV reaches its maximum peak during the winter season, for some during the months of January–February [26,40], December–February [29,37,38], January–March [32], and February–May [42].

### 3.5. Environmental Risk Factors

A negative correlation is found between cold temperatures and the number of RSV positive children as well as the number of hospitalizations [37]; and a positive correlation if there is a higher relative humidity [37]. The spikes of contagion by RSV are also related to certain atmospheric pollutants such as benzene [37].

The environmental home conditions, such as the use of heating with stoves, are related to the number of cases of RSV infection, finding that these children present more serious episodes. In addition, exposure to tobacco is also considered a risk factor [26,29,40].

### 3.6. Another Risk Factors

In relation to the place of residence, there is an increase in the number of RSV hospitalizations in children living in urban areas and in the crowded household population [26]. Those children who attend kindergarten [30] have a higher rate of RSV infection. Moreover, the risk increases the longer the length of hospital stay [43].

### 3.7. Meta-Analysis Prevalence Estimation

**Figure 2:**
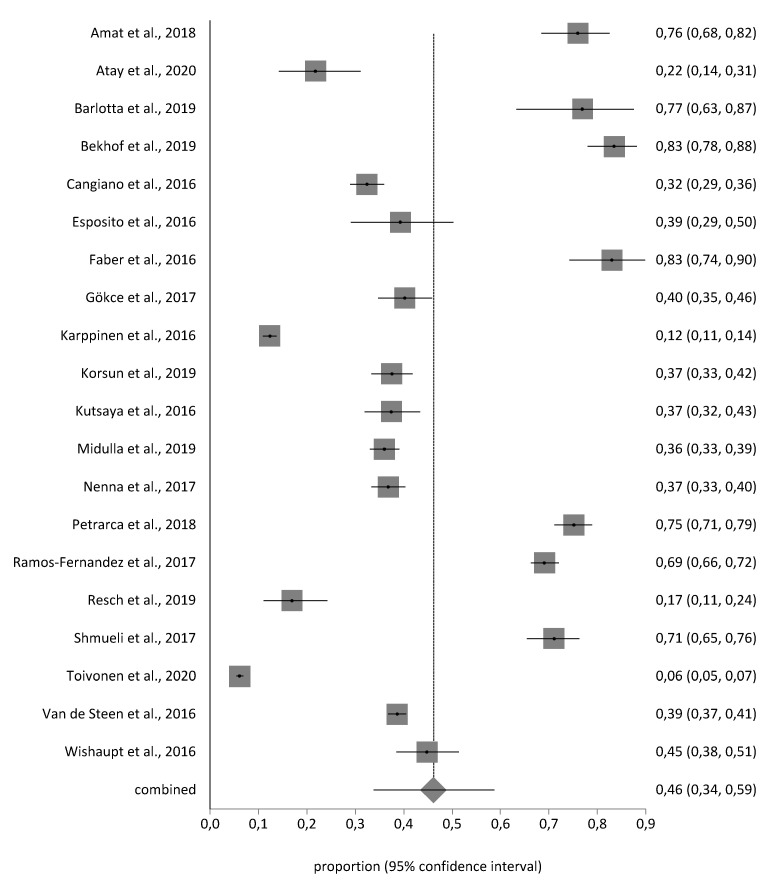
Forest plot of RSV prevalence.

The sample of the meta-analysis was n = 16,115 children. The meta-analytical estimation of RSV was 46% (95% CI 34–59%). The I^2^ value was 99.4% indicating a high heterogeneity. Egger test did not show publication bias and no study was removed after the sensitivity analysis. The forest plot is shown in Figure 2.

## 4. Discussion

The prevalence of RSV infection in children from WHO European countries was 46% (95% CI 34–59%). This percentage is similar to that of other continents such as Latin America, with an RSV prevalence of 41.5% (95% CI 32–51.4%) [45]. Other authors find a global positive rate of RSV infection lower than that of this study, as is the case in Africa with a prevalence of 14.6% (95% CI 13–16.4%) [46] or China with 16% (95% CI 12.9–19.6%) [47]. Even studies carried out in European countries such as France find a lower prevalence of positive RSV of 12–18% [48] and in the US the percentages of RSV infection in healthy children are only 1.8% [49].

Regarding age, we found that RSV infection was detected more frequently in children up to 3 years of age. These results are similar to those of other studies that confirm a significantly higher rate in children under 3 years of age (19.5%) compared to children over 3 years of age (5.6%) [47]. Even multicenter studies find that in children under 6 months the prevalence is 50% and in children under 2 years of age 88%, a fact that corroborates the high burden of RSV in children < 2 years, especially in infants < 6 months [50,51]. The younger the age, the greater the risk of RSV infection, as corroborated by several authors, indicating a greater risk especially in children between 3 to 5 months [48,52,53], although other studies point out that RSV-associated hospitalizations reach their maximum peak in children under 3 months [54]. Other authors suggest a higher percentage of infection in children 0 to 12 months of age [45,54,55,56,57,58].

Gender influences RSV infection, boys have a higher risk of severe RSV infection compared to girls [48,59]. Some studies that have investigated these differences in greater depth indicate an infection ratio of two boys for every girl, the difference in the sex ratio being the highest in the first months of life [59].

Episodes of infection usually occur between November and March, with a peak in January–February [51]. Even some studies when analysing the percentage of RSV infection and its relationship with the month of birth, find a low prevalence for children born in April of 5.7%, being higher in children born in November, of 49.6% [60].

Climatic factors also contribute to promoting RSV infection. Like our results, many studies have highlighted that colder temperatures during the months with the highest RSV prevalence are associated with a greater probability of hospitalization for RSV [51,57]. Furthermore, the positivity of RSV is also higher in environmental conditions of high relative humidity, high atmospheric pressure and even wind speed [51,61].

Other authors, as in this study, consider the importance of exposure to tobacco smoke as a risk factor for RSV infection, aggravating the severity of bronchiolitis or the risk of other acute lower respiratory tract infections [62], although others do not find this association [51,63].

RSV positivity is higher in children with characteristics such as low socioeconomic status and the length of the children’s hospital stay also has a negative influence [51]. In this review, we did not find a relationship between maternal aspects and RSV infection, while other studies indicate a short duration of breastfeeding as a risk factor as well as being born by spontaneous vaginal delivery [51].

The high rate of RSV infection and its negative consequences for the health of children makes it necessary to analyse the existing prevalence, as well as the associated risk factors to prioritize and early identify children at high risk of developing infection associated with RSV [64]. However, most of the research is focused on analysing RSV-associated infection without providing data on its classification according to age.

Hospital admissions for respiratory infections due to RSV remain a significant health problem among young children. However, underreporting, misclassification, and lack of national guidelines regarding RSV diagnostic testing indicate that statistics are rarely sufficient to estimate the incidence or prevalence of RSV-associated disease, which implies more studies in order to know real data [64]. Furthermore, few studies have been conducted to assess the burden of RSV infection in healthy children. A global report of the monthly activity of these viruses is needed to develop public health strategies and programs for their control.

Further efforts are needed to find new strategies to reduce the RSV. Thus, developing measures in order to reduce children’s exposure to indoor and outdoor pollution and identifying additional environmental factors, would contribute to reducing RSV hospitalization and morbidity and mortality.

### Limitations

First, the number of studies is low because not all studies reported the prevalence of RSV or its risk factors. Furthermore, many studies contemplated different respiratory diseases in the global population without analysing the different age groups. Second, the analysis of risk factors has been performed individually, lacking multivariate analysis.

Finally, although the inclusion criteria were established to find studies with similar populations, there was high heterogeneity between the studies. It can be explained by the diversity of countries, where the health-seeking behaviour of populations is likely to vary depending on different cultural contexts. 

## 5. Conclusions

To our knowledge, this is the first meta-analysis to address the prevalence of RSV infection and associated factors in children under 5 years of age in the WHO European Regions. A 46% prevalence of RSV was observed. Among the main risk factors are age, male gender, winter season, and environmental factors such as cold temperatures, higher relative humidity, high concentrations of benzene, exposure to tobacco, and living in urban areas. All of them are potentially predictive factors to prevent the development of RSV infection and, therefore, should be taken into account when establishing prevention strategies. RSV infection in children, as the most vulnerable group, deserves more research, particularly at promoting the provision of a vaccine for this population group.

## Figures and Tables

**Table 1:** Characteristic of included studies (*n* = 20).

Author, (Year), Country.	Study Design/Period	Age	Diagnostic Laboratory Procedure	Sample Size Positive Cases	Setting	Prevalence	EL/RG
Amat et al. [25] 2018 France	Cohort study 2011–2012	<3 years	Antigen tests/FEIA	*N* = 154 *n* = 117	Outpatients and hospitalized children	75.9%	2b/B
Atay et al. [26] 2020 Turkey	Cross sectional study 2011–2012	2 months–2 years	PCR	*N* = 101 *n* = 22	Inpatient or outpatient clinics	21.78%	2c/B
Barlotta et al. [27] 2019 Italy	Cohort study 2013–2014	<1 year	PCR	*N* = 52 *n* = 40	Inpatient ward	76%	2b/B
Bekhof et al. [28] 2019 Netherlands	Cohort study 2012–2016	<2 years	PCR	*N* = 218 *n* = 182	Inpatient ward	83.4%	2b/B
Cangiano et al. [29] 2016 Italy	Cross sectional 2004–2014	<1 years	PCR	*N* = 723 *n* = 234	Inpatient ward	32.36%	2c/B
Esposito et al. [30] 2016 Italy	Cohort study 2013	<18 months	PCR	*N* = 89 *n* = 35	Inpatient ward	39.3%	2b/B
Faber et al. [31] 2016 Netherlands	Cohort study	<13 months	PCR	*N* = 100 *n* = 83	Inpatient ward	83%	2b/B
Gökçe et al. [32] 2017 Turkey	Cross sectional study 2013–2016	<2 years	PCR	*N* = 316 *n* = 127	Inpatient ward	40.1%	2b/B
Karppinen et al. [33] 2016 Finland	Cohort study 2005–2012	<2 years	PCR	*N* = 2275 *n* = 279	Inpatient ward	12.26%	2b/B
Korsun et al. [34] 2019 Bulgaria	Cohort study 2015–2018	<5 years	PCR	*N* = 515 *n* = 193	Inpatient or outpatient patients	37.5%	2b/B
Kutsaya et al. [35] 2016 Finland	Cohort study 2009–2013	<1 years	PCR	*N* = 291 *n* = 109	Inpatient patients	37.45%	2b/B
Midulla et al. [36] 2019 Italy	Cohort study 2005–2012	<1 years	PCR	*N* = 998 *n* = 359	Inpatient ward	35.97%	2b/B
Nenna et al. [37] 2017 Italy	Cohort study 2004–2014	<1 years	PCR	*N* = 723 *n* = 266	Inpatient ward	36.79%	2b/B
Petrarca et al. [38] 2018 Italy	Cross sectional 2004–2016	<1 years	PCR	*N* = 486 *n* = 365	Inpatient ward	75.1%	2b/B
Ramos-Fernández et al. [39] 2017 Spain	Case-control study2010–2015	<6 months	Antigen tests	*N* = 1006 *n* = 695	Inpatient ward	69%	1b/A
Resch et al. [40] 2019 Austria	Cohort study 2005–2015	39–42 weeks	PCR	*N* = 136 *n* = 23	Inpatient ward	16.9%	2b/B
Shmueli et al. [41] 2017 Israel	Cohort study 2012–2013	<2 years	Antigen tests/FEIA	*N* = 286 *n* = 203	Inpatient ward	70.9%	2b/B
Toivonen et al. [42] 2020 Finland	Cohort study 2008–2010	<2 years	Antigen tests/PCR	*N* = 4728 *n* = 289	Inpatient or outpatient clinics	6%	2b/B
Van de Steen et al. [43] 2016 Central and Eastern Europe countries ^1^	Cohort study 2009–2011	<1 years	Antigen tests	*N* = 2677 *n* = 1034	Inpatient ward	38.62%	2b/B
Wishaupt et al. [44] 2016 Netherlands	Cohort study 2011–2014	<3 months	PCR	*N* = 241 *n* = 108	Inpatient ward	40.14%	2b/B

*Note*: EL = Evidence level; FEIA = fluorescence immuno-enzymatic assay; ICU = intensive care unit; PCR = polymerase chain reaction; RG = Recommendation grade; RSV-IC = RSV immunochromatographic detection. ^1^ Central and Eastern Europe Countries: Estonia, Lithuania, Hungary, Slovenia, Croatia, Serbia, Bosnia/Herzegovina, Bulgaria, Czech Republic, Slovakia, Romania, and Ukraine.

**Table 2:** Main risk factors associated with RSV infection.

Author, (year)	Risk Factors
Atay et al. [26], 2020	Male gender (OR 1.73, 95% CI 1.0–5.7%, *p* = 0.302) Crowded household population (OR 1.73, 95% CI 1.0–5.7%, *p* = 0.046) Heating stoves (OR 0.31, 95% CI 0.1–0.98%, *p* = 0.025) Urban cities (*p* < 0.001) Exposure to smoking (*p* = 0.001) Peak February
Cangiano et al. [29], 2016	Younger (*p* < 0.001) Peak December–February Maternal smoke during pregnancy (*p* = 0.036)
Esposito et al. [30], 2016	Age (OR 6.3, 95% CI 1.4–33.9%, *p* = 0.02) Age ≥ 5 months old (children 5–8 months old and 9–16 months old vs. children 0–4 months old: *p* = 0.03 and *p* = 0.003, respectively) Attended day care (*p* = 0.001) Birth date (OR 2.7, 95% CI 1.1–6.7%, *p* = 0.03) Male gender (OR 2.3, 95% CI 1.0–5.7%, *p* = 0.06)
Gökçe et al. [32], 2017	Peak January–March Younger (<6 months)
Karppinen et al. [33], 2016	Male (OR 1.06, 95% CI 0.57–1.99%, *p* = 0.85) Socioeconomic status (OR 0.78, 95% CI 0.39–1.56%, *p* = 0.48)
Korsun et al. [34], 2019	Youngest age group: <6 months (50%), followed by 6–11 months (38.5%) Of the RSV-positive children 58.5% were under 2 years of age: 61% were boys and 39% were girls (*p* = 0.5773)
Kutsaya et al. [35], 2016	RSV seroprevalence increased from 37% at age 1 year to 68% at age 2 years, and to 86% at age 3 years
Midulla et al. [36], 2019	Younger (*p* < 0.0001) Lower body weight at admission (*p* = 0.005) Born in winter
Nenna et al. [37], 2017	The number of RSV-positive infants correlated negatively with cold temperature (r = −0.46, *p* < 0.001), and positively with higher relative humidity (r = 0.36, *p* < 0.001) Peak December–February
Petrarca et al. [38], 2018	No relation to exposure to smoke, breastfeeding, birth weight and sex
Ramos-Fernández et al. [39], 2017	Male (OR 4.27, 95% CI 1.14–15.93%, *p* = 0.03) Low birth weight (<3rd percentile) (OR 5.53, 95% CI 0.93–32.97%, *p* = 0.06)
Resch et al. [40], 2019	No relation to smoking during pregnancy Peak January
Toivonen et al. [42], 2020	Of all RSV infections, 10% occurred before 3 months of age, 16% at 3–5 months of age, 32% at 6–11 months of age, and 42% at 12–24 months of age Peak February–May
Van de Steen et al. [43], 2016	Duration of hospitalization (*p* < 0.001)

## Data Availability

Data available in request to correspondence author.
